# Microwave Digital Twin Prototype for Shoulder Injury Detection

**DOI:** 10.3390/s24206663

**Published:** 2024-10-16

**Authors:** Sahar Borzooei, Pierre-Henri Tournier, Victorita Dolean, Claire Migliaccio

**Affiliations:** 1 Laboratoire d’Electronique Antennes et Télécommunications (LEAT), Université Côte d’Azur, 06000 Nice, France; 2 Laboratoire Jean Alexandre Dieudonné, Université Côte d’Azur, 06000 Nice, France; 3 Laboratoire Jacques-Louis Lions (LJLL), CNRS, Inria, Sorbonne Université, 75005 Paris, France; pierre-henri.tournier@sorbonne-universite.fr; 4 Department of Mathematics and Computer Science, Eindhoven University of Technology, P.O. Box 513, 5600 MB Eindhoven, The Netherlands; v.dolean.maini@tue.nl

**Keywords:** machine learning, numerical modeling, microwave sensing system, tendon injury, SVM classification

## Abstract

One of the most common shoulder injuries is the rotator cuff tear (RCT). The risk of RCTs increases with age, with a prevalence of 9.7% in those under 20 years old and up to 62% in individuals aged 80 years and older. In this article, we present first a microwave digital twin prototype (MDTP) for RCT detection, based on machine learning (ML) and advanced numerical modeling of the system. We generate a generalizable dataset of scattering parameters through flexible numerical modeling in order to bypass real-world data collection challenges. This involves solving the linear system as a result of finite element discretization of the forward problem with use of the domain decomposition method to accelerate the computations. We use a support vector machine (SVM) to differentiate between injured and healthy shoulder models. This approach is more efficient in terms of required memory resources and computing time compared with traditional imaging methods.

## 1. Introduction

The shoulder is the most mobile joint in the body, allowing rotation across multiple axes, with some capable of full 360° rotation, as well as enabling arm elevation and overhead reaching. This mobility is facilitated by the rotator cuff, a complex group of muscles and tendons. With repetitive movements, the rotator cuff wears out, eventually leading to rotator cuff tears (RCTs). This injury most commonly occurs with aging, but it also affects athletes and individuals in professions that involve frequent shoulder movements, such as manual labor or cleaning, making it one of the most prevalent shoulder injuries. According to [1], approximately 2 million people in the U.S. consult their physicians each year for this condition. RCTs can advance to more serious conditions over time, reinforcing the relevance of early detection. In [2], the prevalence of rotator cuff tears in the general population was reported to be 22.1%. Magnetic resonance imaging (MRI) is the gold-standard imaging technique. However, its use is restricted to imaging centers, and it does not always provide accurate depictions of the presence and severity of tears [3]. In [4], it was reported that the overall accuracy for detecting RCTs of different sizes with the use of MRI is 87%.

With the occurrence of RTCs, synovial fluid (SF) aspirates locally in the injury area [5,6]. This accumulation of SF changes the dielectric properties of the shoulder joint [7]. This change makes microwave imaging (MWI) a credible alternative to MRI which we need to investigate. The portability and costs of MWI systems make them ideal candidates for fast and early diagnosis. At this stage, the main issue is detecting the presence of RCTs. As discussed with physicians, this would be the first step, and if the detection is positive, then the patient will undergo an advanced imaging modality such as MRI in order to evaluate the size and location of the RCT. In [8], we introduced an alternative low-cost, portable and non-invasive electromagnetic imaging (EMI) system for the on-site diagnosis of RCTs. At that time, no EMI system for the shoulder existed, (To the best of our knowledge, this remains true.) and thus we had to start from scratch. To save time and resources during the initial design phase, we developed a virtual model of the shoulder and an imaging system to study and optimize the EMI system, as described in [8]. This model is the first step toward a microwave digital twin prototype (MDTP).

The concept of the digital twin (DT) was originally proposed by Michael Grieves at the University of Michigan for monitoring product lifecycle management. This involves creating a virtual model of a physical system, which is continuously updated with real-time data from the existing physical system. DTs are not intended for system design. However, in [9], the same authors introduced the digital twin prototype (DTP), which exists in virtual space and is to be used in what the authors referred to as the creation phase. Since 2002, DTs have been widely used and developed for Industry 4.0 applications [10,11,12]. In the healthcare sector, a comprehensive review of Digital Twin for Health (DT4H) can be found in [12]. A wide range of applications was already investigated, including detecting and monitoring cardiac pathologies, diabetes, breast or oropharyngeal cancers and Alzheimer’s diseases. DT4H often incorporates machine learning (ML) in order to enhance the performance of illness detection, as exemplified in [13] with COVID-19. Quite recently, DTs have been efficiently used for microwave ablation [14] and imaging purposes [15]. In this paper, we introduce the concept of a microwave digital twin prototype as a virtual system which mimics the physical one and is capable of predicting the presence of RCTs. The model not only includes the anthropomorphological model of the shoulder (whether it is injured or not) but also the imaging system and uncertainties due to its use, like noise, positioning errors and errors due to RCTs themselves, like the synovial fluid’s variation, which depends on the RCT’s severity.

Compared with our previous work [8], we aim to improve and systematize the detection of RCTs. Thus far, we have been solving an inverse problem for detecting the presence of RCTs. This process is time-consuming, requires extensive computing resources and is therefore not compatible with a large number of case studies. As an example, the final design consists of 32 ceramic ($\varepsilon_{r}=59$) loaded, open-ended waveguides. It requires 11 min and 27 s for image reconstruction of one shoulder model with the use of 480 computing cores. These amounts of resources may not always be available and can limit the practical use of the device in the real world. In this paper, we aim to address this issue through the use of ML algorithms.

The rise of ML has led to the development of valuable tools in various medical applications, such as predicting sports injuries [16], simplifying medical imaging processes [17] and advancing stroke medicine [18]. Further, combining microwave imaging systems with ML algorithms has significantly improved stroke detection, stroke type classification and localizing affected areas [19,20,21].

Dataset gathering is a crucial component of machine learning algorithms, particularly in medical applications, but it presents numerous challenges and limitations [22]. For example, insufficient or biased data can result in poor generalization, which highly affects the algorithm’s accuracy in making predictions or diagnoses. To enhance generalization, large and diverse training datasets are necessary. Moreover, the effectiveness of ML algorithms heavily relies on the quality and quantity of the data. However, in the real world, obtaining data from patients involves privacy and authorization challenges and is a time-consuming process. Furthermore, the limited available training data significantly impacts the performance of the classifiers. To address this issue, generating synthetic data through numerical simulations or various computer algorithms has emerged as a promising solution in recent years [23,24]. In [25], numerical simulations of a system were performed to investigate how integrating mathematical models with experimental datasets could enhance classification performance. It is important to note that while the use of synthetic data can enhance continual and causal learning, it also carries the risk of introducing biases [26]. This emphasizes the importance of generating a reliable dataset.

In this work, we use numerical modeling to generate a generalizable dataset of scattering parameters. A parametric study is conducted considering four main categories, which are outlined in Table 1. For the classification of injured and healthy models, we utilize a supervised machine learning support vector machine (SVM).

Note that the key indicator in differentiating healthy and injured shoulder joints is the presence of RCTs, because this is the most challenging case. The mean aspirate volume of SF is reported to correlate with the size of the tear. This volume for the small tears is 1.46±1.88 mL, while that for medium tears is 3.04±2.21 mL and that for large tears is 6.60±3.23 m [6]. In this study, we will consider the presence of a small tear in an injured shoulder model, as it is the most difficult tear to detect. This paper is structured as follows. Section 2 presents the numerical modeling framework, including the numerical modeling of the system, its properties and our methods for data generation and classification analysis using an SVM. Section 3 discusses the numerical results for various scenarios, and the conclusions are provided in Section 4.

## 2. Mathematical Framework

### 2.1. Antropomorphic Model of the Shoulder

**Figure 1:**
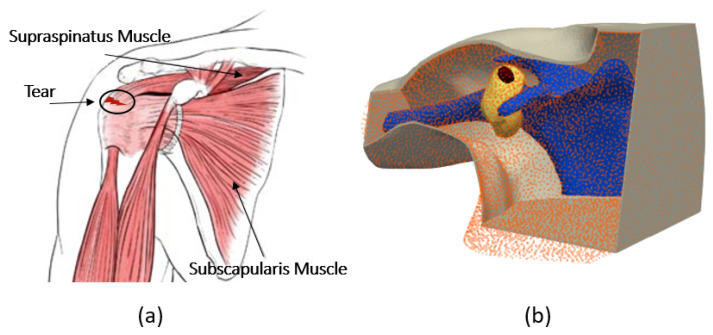
(**a**) Anatomy of the shoulder. (**b**) Numerical model of the shoulder.

Accurate finite element modeling of the shoulder is first step to conducting a reliable numerical study to detect RCTs. Figure 1a shows a view of the anatomy of the shoulder, and Figure 1b shows an anthropomorphic model of different tissues. To build this realistic shoulder model, we used computer-aided design (CAD) models for the shoulder profile, scapula and humerus bones. These CAD models were achieved through a library of 3D anatomy models (https://www.plasticboy.co.uk/store/index.html, accessed on 17 February 2023). The rotator cuff tendons were modeled to surround the shoulder joint, and the skin with a thickness of 2 mm was modeled to surround the shoulder structure. The injury was modeled by an ellipsoid with a volume of 1.4 mL filled with SF, which represents a small RCT [6]. Note that modeling an injury as an ellipsoid in medical imaging is common because it captures the asymmetrical and irregular expansion typical of many biological injuries, especially in soft tissues. The remaining space in the shoulder joint was filled with muscle. Note that in the healthy shoulder model, the electrical properties of the injury area are changed to those of the muscle. In our simulations, we assigned complex permittivity values of each tissue at 1 GHz, as reported in Table 2. In this Table, the SF value is based on our recent work in [8], and the values of other tissues are based on the reference websites (http://niremf.ifac.cnr.it/tissprop/, accessed on 1 September 2023 and https://itis.swiss/virtual-population/tissue-properties/, accessed on 1 September 2023). We considered the value of the complex permittivity of the matching medium to be constant for all of the simulations and equal to that of the muscle (54.8−17.43i). If we considered the value of the matching medium as the reference, then the wavelength in this medium was λ=4.05 cm.

### 2.2. Numerical Model of the System

**Figure 2:**
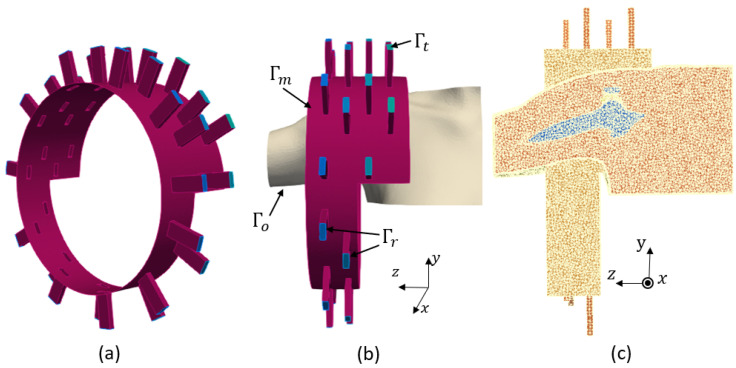
(**a**) Imaging system. **(b**) Boundary conditions. (**c**) Finite element mesh.

In this work, we use the optimized sensing system which was designed in our previous work [8], shown in Figure 2a. This configuration was designed to detect the smallest RCT while using the minimum number of antennas through a differential imaging method. It consists an array of 32 ceramic ($\varepsilon_{r}=59$) loaded, open-ended waveguides which illuminate the shoulder from different angles. This multi-view approach helps in gathering comprehensive and accurate data to represent the effect of the internal tissues of the shoulder on the scattering parameters. The waveguides are arranged on two metallic, fully circular and two metallic half-circle layers. The width the rectangular waveguides is 2.1 cm, and their height is 0.75 cm. Their frequency bandwidth is 0.93–1.85 GHz. We chose to work with a single frequency because this drastically simplified the requirements for the final system (no need to have wide band components) and the measurement time. We selected a 1 GHz operating frequency because it led to reaching a good trade-off between the resolution and penetration depth.

The two sides of the imaging chamber are open to allow the insertion of a real shoulder, as shown in Figure 2b. A cross-section of the finite element three-dimensional (3D) mesh of the complete system, including the imaging system and the shoulder, is shown in Figure 2c. In this mesh, the maximum diameter of the mesh cells is $n_{\lambda }=\lambda /9$, which yields h= 0.45 cm. First-order finite element discretization of this problem yields 1,891,259 as the number of degrees of freedom. We used the open source FreeFEM software (v.4.13) for the forward modeling of our problem.

The generated 3D domain (Ω), including the sensing system and the shoulder, is a heterogeneous, dissipative non-magnetic medium of a complex permittivity $\varepsilon_{r}=\left(\varepsilon_{r}^{\prime }-\frac{\sigma j}{\omega \varepsilon_{0}}\right)$, where $\varepsilon_{r}^{\prime }$ is the relative permittivity of each tissue, $\varepsilon_{0}$ is the permittivity of free space, σ is the conductivity and ω is the angular frequency. Each transmitting antenna emits a time periodic signal, where E(x) is the complex amplitude of the associated electric field $e\left(x,t\right)=ℜ(E\left(x\right)e^{i\omega t})$ at the space variable x. We found E(x) as the solution for each transmitting antenna by solving the boundary value problem defined in Equation (1):

(1) $$\left\{\begin{array}{cc} \nabla \times \left(\nabla \times E\right)-k^{2}E=0, & \mathrm{in}\;\Omega \\ \nabla \times E\times n+i\beta n\times (E\times n)=0 & \mathrm{on}\;\Gamma_{r}\\ \nabla \times E\times n+ikn\times (E\times n)=0 & \mathrm{on}\;\Gamma_{o}\\ \nabla \times E\times n+i\beta n\times (E\times n)=g & \mathrm{on}\;\Gamma_{t}\\ E\times n=0 & \mathrm{on}\;\Gamma_{m} \end{array}\right.$$

where β is the propagation constant along the waveguide, n is the unit outwardly normal to the boundaries, $k=\omega \sqrt{\varepsilon_{r}\varepsilon_{0}\mu_{0}}$ is the complex wavenumber of the inhomogeneous medium and $\mu_{0}$ is the permeability of the free space. We define the excitation term as $g=2i\beta E^{TE_{10}}$. This imposes an incident wave which corresponds to the excitation of the dominant transverse electric mode (TE_10_) of the active waveguide. The boundaries are shown in Figure 2b and defined as follows. Here, $\Gamma_{r}$ presents the ports of the receiving waveguides, $\Gamma_{o}$ presents the open sides of the chamber and the boundaries of the shoulder profile which are outside of the chamber, $\Gamma_{t}$ presents the ports of the transmitting waveguide, and $\Gamma_{m}$ presents the metallic surfaces of the chamber and the waveguides. Through solution of the boundary value problem for each transmitting antenna, we can compute the scattering matrix ${\left(S_{ij}\right)}_{1\leq i,j\leq 32}$ using Equation (2):

(2) $$S_{ij}=\left\{\begin{array}{c} \frac{\int_{\Gamma_{r}}\bar{E^{j}}\cdot E^{TE_{10}}}{\int_{\Gamma_{r}}{\left|E^{TE_{10}}\right|}^{2}}\;\;\;i\neq j,\\ \frac{\int_{\Gamma_{r}}\bar{E^{j}}\cdot E^{TE_{10}}}{\int_{\Gamma_{r}}{\left|E^{TE_{10}}\right|}^{2}}+1\;\;i=j, \end{array}\right.$$

where *j* represents the transmitting port and *i* represents the receiving port.

### 2.3. SVM

We chose an SVM for our classification tasks due to its effectiveness in binary classification problems, which aligns with the nature of our dataset, where we sought to detect whether the RCT was present or not. Given that our problem was straightforward, and the dataset was relatively small, an SVM is particularly well suited as it offers a robust and interpretable approach. Its straightforward implementation and tuning processes also enabled us to obtain dependable classification results without the need for more complex algorithms.

This supervised machine learning algorithm is designed to recognize patterns and relationships between features and the target variable within a dataset. This process, known as training or fitting, aims to prepare the algorithm to accurately predict labels for new, unseen data [27]. The SVM algorithm involves three main steps: training, validation and testing. The training and validation phases together form the development phase. During the training step, the model learns the characteristics of the data to determine the best boundaries for class separation. The validation step involves using part of the training set to evaluate the model’s performance and make necessary adjustments to enhance its effectiveness on unseen data. Finally, the test phase uses an entirely new dataset to assess the model’s overall performance.

The performance of an SVM is highly dependent on the optimal selection of hyperparameters. For any type of SVM, the hyperparameter *C* needs to be optimized as it controls the margin which separates the two classes. A linear SVM kernel is used for linearly separable data, whereas more complex kernels, like radial basis function (RBF) kernels, are applied when the data are not linearly separable. For nonlinear kernels, the hyperparameter γ shapes the decision boundary and must also be optimized. This paper employs the grid search (GS) technique to choose the appropriate kernel method and determine the optimal hyperparameters. Note that we avoided overfitting for all the scenarios through a cross-validation technique. A good choice for the C parameter in SVM classification allows a generalizable boundary which performs well on training, validation and test sets.

### 2.4. Evaluation Metrics

A confusion matrix is frequently used to evaluate the performance of binary classification problems. In this work, samples obtained from healthy shoulder models are labeled as +1, while samples from injured models are marked as −1. It is a cross-table, demonstrated in Table 3, which captures the occurrences of actual classifications versus predicted classifications. In this table, TN is true negative, TP is true positive, FP is false positive, and FN is false negative. These are numbers in sets of {0,1,…,N}.

The table assumes that there were FN+TP healthy samples, of which only TP were correctly recognized as healthy, whereas FN were incorrectly recognized as injured. Similarly, there were TN+FP injured models, of which only TN were correctly identified as injured, while FP were identified as healthy by mistake. To assess these classifications, we calculated the accuracy (acr) as the proportion of correctly classified samples out of the total number of samples, the sensitivity (sens) as the probability of correctly recognizing the healthy samples or true positives, and the specificity (spec) as the probability of correctly recognizing the injured samples or true negatives.

### 2.5. Dataset Processing

The different steps for numerically generating a sample with the use of the imaging system are illustrated in Figure 2. We needed to solve the boundary value problem of Equation (1) to determine the scattering matrix. To calculate the scattered field, we first computed the scattering coefficients for the domain filled with only the homogeneous matching medium ($S_{ij}^{\mathrm{empty}}$) and then subtracted these from the scattering coefficients obtained with the shoulder present ($S_{ij}^{\mathrm{full}}$). The difference between these two matrices is a matrix $S_{ij}$ of scattering parameters 32×32=1024 in size. Then, to simulate a realistic experimental condition, we generated $S_{ij}^{\mathrm{syn}}$ by adding multiplicative white Gaussian noise independently to the real and imaginary parts of each $S_{ij}$.

**Figure 3:**
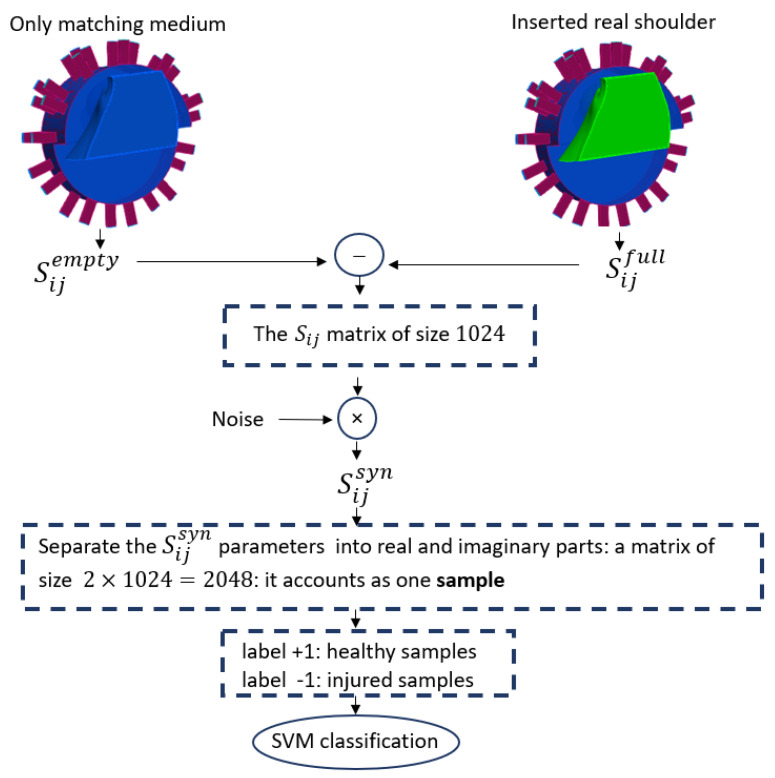
The workflow of SVM classification.

We built the complex nature dataset using the separate values of the real and imaginary parts of $S_{ij}^{\mathrm{syn}}$, which doubled the dimension of the scattering matrix to 2048 in size. This vector of synthetic data was introduced as an sample for the ML algorithm. Figure 3 shows the steps of data preparation in our method.

The partitioning in the development set was as follows: 90% for the training set and 10% for the validation set.

Throughout this paper, we will show the three most significant eigenvectors computed from the PCA applied to the training and test datasets separately. Note that PCA was used to visually evaluate the differences and similarities between the classes [28] and not for classifying. Indeed, considering that the SVM could work directly with the original features, and to avoid loss of information, we chose to directly classify the raw data. In the next sections, we will give a detailed description of the generated dataset for different scenarios.

## 3. Numerical Results

In this section, we introduce different scenarios to conduct a feasible numerical study of classifying healthy and injured shoulder models. We conducted our study using grid search and a linear SVM to ensure faster testing. This helped us establish baseline performance, gain insights into the data and efficiently explore hyperparameter spaces through grid search. Once we had a solid understanding of the data’s behavior and potential patterns, we could consider more complex models like a nonlinear SVM for further refinement.

### 3.1. Influence of Noise

The classification of injured and healthy shoulder models for the generated synthetic data with different noise levels is studied in this section. Studying the influence of extremely high noise levels, especially close to the Shannon limit (−1.6 dB), is crucial for evaluating model robustness, understanding performance boundaries and ensuring reliability in practical, noisy environments.

To consider a more realistic scenario, the noise levels among the training and test datasets as well as the healthy and injured samples were different. The detailed introduced noise levels for each category are mentioned in Table 4. For the test set, we considered six different noise levels, and for training set, we considered five different noise levels. Then, for each noise level, we generated samples with 36 different seeds to build a large dataset. The number of samples for each category is presented in Table 5.

Let us use the GS method for optimization of the hyperparameter *C*, with the results reported in Table 6. We can see that when C= 6,000,000, an accuracy of 100% was achieved.

### 3.2. Dehydration Error in Complex Permittivity

In [29], it was reported that the dielectric properties can vary as a function of time at different temperatures due to dehydration. This relative change was measured to be 9%, and we called it $hd_{err}$. The effect of $hd_{err}=\pm 9\%$ on the complex permittivity is reported in Table 7. Looking at this table, we can see that the contrast between the dielectric properties of the SF and the muscle could become quite small, specifically for cases where the dehydration error for the muscle was bigger than the one for the SF.

Considering that this contrast is the key element of differing between healthy and injured models, it is crucial to study the effect of this parameter. To build datasets for both the healthy and injured shoulder models, we included three different variations of the muscle. Note that for the case of the injured model, we repeated the simulations in three groups for each value of SF to be able to distinguish the dataset. For both the healthy and injured models, we introduced seven different noise levels from 23 dB to 10 dB and 48 seeds for each case. Table 8 explains the method of data generation for this scenario. The matrix $\left[\begin{array}{c} 1\\ 1\\ 1 \end{array}\right]$ for the injured model held for each group of generated datasets with different values of $hd_{err}$ for the SF. Then, we separated 900 randomized samples for the training set and 108 samples for the test set for each category for the healthy and injured models.

We repeated the classification three different times with different SF values. The results are reported in Table 9. This demonstrates that for all three SF values, we could have 100% accuracy in classifying the healthy and injured models when possible different values of $hd_{err}$ for the muscle were also included in the dataset.

### 3.3. Positional Error of the Phantom

The location of the shoulder in the imaging system can vary due to the patient’s body habits, which can impact the values of the computed scattering parameters. In this section, the objective is to determine whether it is possible to detect injuries when the locations of the phantoms in the imaging system differ between the training and test datasets. In addition, we include all effects which have been previously introduced, such as noise and dehydration. For each location, as far as the permittivity was concerned, we introduced the three values of the muscle’s permittivity which were used in the former section. For the injured model, we also included three values for the SF. In addition, we took four different levels of noise comprised between 10 and 23 dB. Finally, we decided on the number of seeds for each model in order to have a balanced number of samples between the two classes in the training dataset; we included 12 seeds for each noise value, whereas we used only 4 for the injured model because the latter already included three different SF values. In this way, we generated 144 samples for the healthy model and 144 samples for the injured model, as shown in Table 10.

#### 3.3.1. Translation Offset

**Figure 4:**
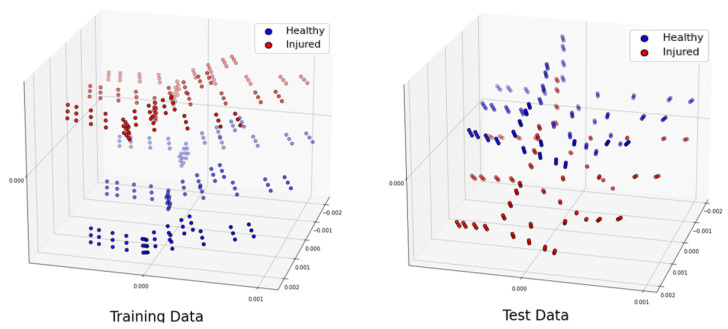
Projection of the three most significant eigenvectors of the training dataset and test dataset when phantoms had a shift of 1 cm along Oz.

**Figure 5:**
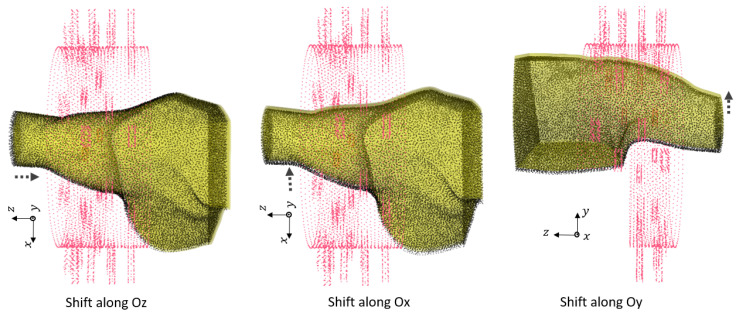
The translation error of of 0.5 cm along different axes between training and test dataset phantoms.

In this section, we investigate the maximum translation offset for which we still obtain optimal classification results. To start, we applied the offset along Oz. The PCA of the 3 most significant eigenvectors of the training dataset and test dataset are shown in Figure 4. The value of C was optimized at C= 5,000,000 with grid search. The translation shift is exemplified in Figure 5 for a value of 0.5 cm. The accuracy was 100% up to a 0.5 cm shift in translation, whereas it fell to 83.33% for 1 cm with a sensitivity of 100% and a specificity of 66.67%. This poor accuracy was due to overfitting, and to solve this problem, we needed to introduce phantoms with small translation steps of 0.5 cm to increase the sample diversity in training. To draw a general conclusion, we repeated this study along Ox and Oy. The results are in line with those obtained for the shift along Oz and show that 0.5 cm is the maximal translation without loss of accuracy.

Note that in the real world, the anatomy of the shoulder limits extensive movement in translation without introducing rotation of the shoulder. Therefore, to simulate the realistic movements of the shoulder, we needed to generate a larger dataset which included various offsets and rotations of the phantoms inside the imaging system. This is investigated in the next section.

#### 3.3.2. Effect of Rotation and Translation Offset

We generated 144 samples based on Table 10 for 31 different locations of the phantoms for both the healthy and injured models. Note that all of the rotations were performed along Oy, and the shifts were performed along Ox and Oz. We chose different locations from the smallest offset (0.5 cm) to the largest possible rotation (θ=25.7°, Δx=−4 cm, Δz=−1 cm). Larger changes could not happen due to limitations on both the shoulder size and the imaging chamber structure. Table 11 shows the total numbers of the dataset. In this study, we included the generated dataset for 30 locations of the phantoms for training (4320 samples), and we excluded one remaining dataset of different positions for the test set (144 samples).

**Figure 6:**
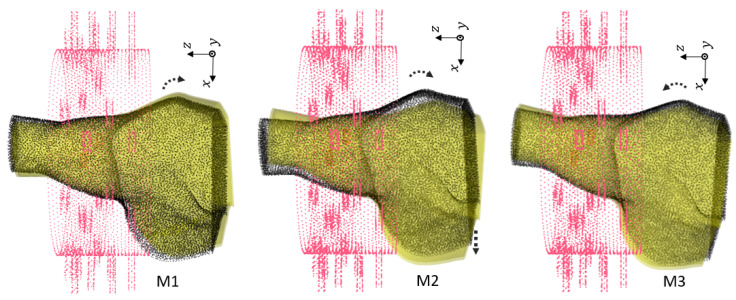
First group of rotations For M1, θ=−3.6°. For M2, θ=−3.6° with shift Δx=−1 cm. For M3, θ=−7.2°.

**Figure 7:**
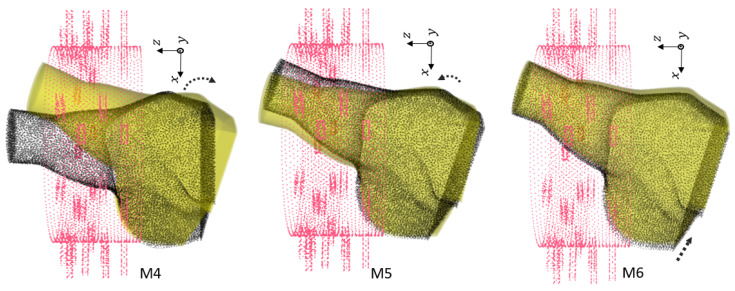
Second group of rotations. For M4, $\theta =-18^{\circ }$. For M5, $\theta =-12^{\circ }$ with shift Δx=−3 cm. For M6, θ=−16.3° with shift Δx=−4.5 cm.

**Figure 8:**
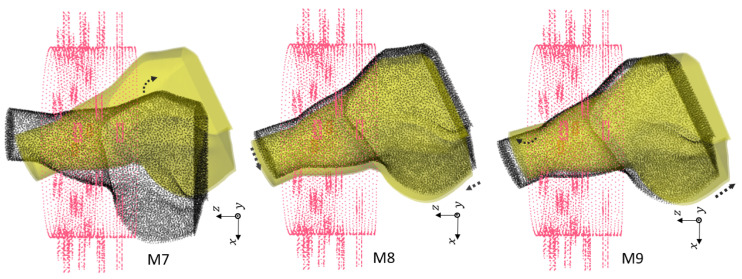
Third group of rotations. For M7, θ=25.7°. For M8, θ=25.7°, Δx=1 cm and Δz=1 cm. For M9, θ=22.5°, Δx=1 cm and Δz=3 cm.

Figure 6, Figure 7 and Figure 8 show the geometries of nine scenarios chosen for their diversity of the change in the position. They were indexed from M1 to M9. Let us remind the reader that both a healthy and injured shoulder were considered for each scenario. For example, M2 was simulated two times: once with the healthy shoulder (called M2-healthy) and once with the injured one (M2-injured).

**Figure 9:**
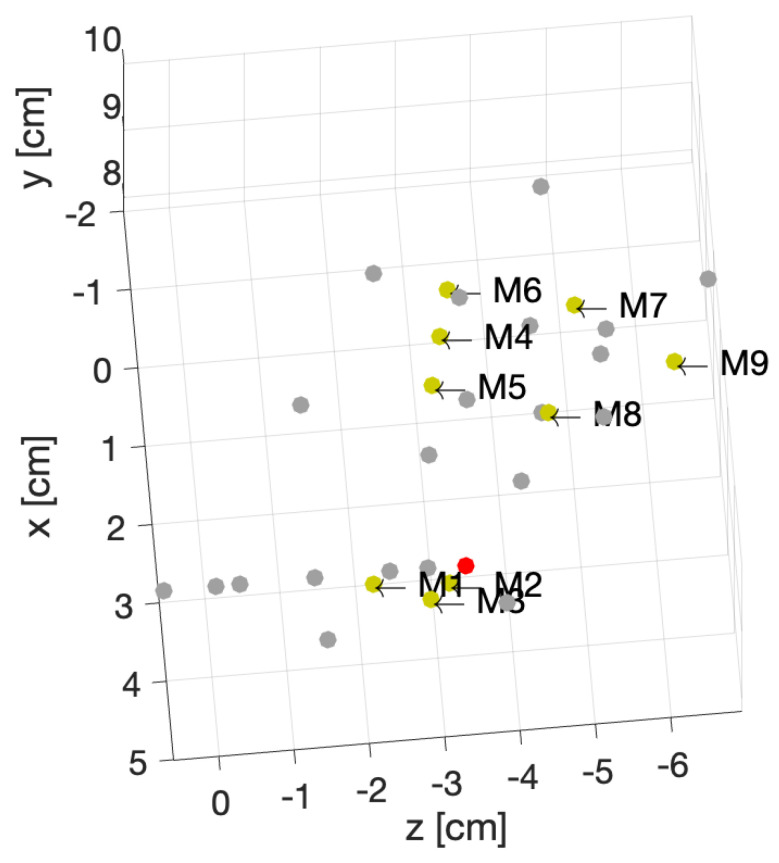
Position of the center of rotations for 31 different phantoms.

To better understand the different included locations of the phantoms, we show the positions of the center of rotation for all 31 scenarios in Figure 9. Nine out of the 31 scenarios were chosen for various tests, and they are specified in this figure with yellow points. The red point accounts for the reference position of the phantom in the absence of translation offset and with no rotation. We can see that these nine chosen points were grouped three by three according to the value of the angle of rotation. For example, the locations of M1, M2 and M3 were slightly different from each other but were nearby scenarios used for the training, which are shown in gray. We had a similar situation for M4, M5 and M6. However, the third group, M7, M8 and M9, had a larger distance from each other.

We examined different scenarios with separation of the test dataset for a specified position, as explained in Table 12. The rest of the samples were included in the training dataset. Note that in this table, the accuracy is mentioned using a linear kernel and C= 600,000,000. We elaborate upon the results for each scenario:

- In first case, the dataset for the M2 phantom for both the healthy and injured shoulders (M2-healthy and M2-injured) was considered for the test. This showed an accuracy of 100% in classifying the healthy and injured models. This accuracy was due to the fact that the M2’s position was close to the phantoms used for training and belonged to the group with a small rotation angle (about 5°).
- The second case (with M2-healthy and M4-injured) is a little bit more challenging because both samples were apart, considering their centers of rotation. However, the similar positions for both existed in the training dataset. M2 belonged to the group with a small rotation (about 5°) angle, whereas M4 belonged to the one with the intermediate rotation angles (about 15°).
- The third case (M7-healthy and M3-injured) was even more challenging compared with the second case because M7 belonged to the group with large rotation angles (about 22°), and M3 belonged to the group with small rotation angles (about 5°). In this case, the classification accuracy dropped to 91% because the position of M7 was apart from the rest of the positions in the training database. Indeed, the distance between M7 and the closest training point was 0.5 cm. Note that all misclassified samples belonged to the class of the healthy shoulder (M7-healthy) incorrectly classified as injured. This is in line with the fact that the position of M7 (which was healthy) was far away from the training phantoms, which made classification more difficult.
- The scenario (M9-healthy and M6-injured) gathered samples belonging to the groups of large and medium rotation angles, respectively, but this time, one sample (M9-healthy) was farther away from the positions that were in the training dataset. The distance between M9 and the closest training point was 1 cm. As in the previous scenario, this led to a poorer classification performance, dropping to 88%, and all misclassified samples were samples from the healthy shoulder incorrectly classified as injured.

As for the translation case, the results obtained from the two last scenarios show that there was a limitation in having a position of the patient which was not previously introduced in the training. We found empirically that this limit was reached when the distance between the center of rotation and the closest point in the training was 0.5 cm. In order to overcome this limitation, we then shuffled all of the samples between the training and test sets. The results are discussed in the next section.

#### 3.3.3. Shuffled Large Dataset

**Figure 10:**
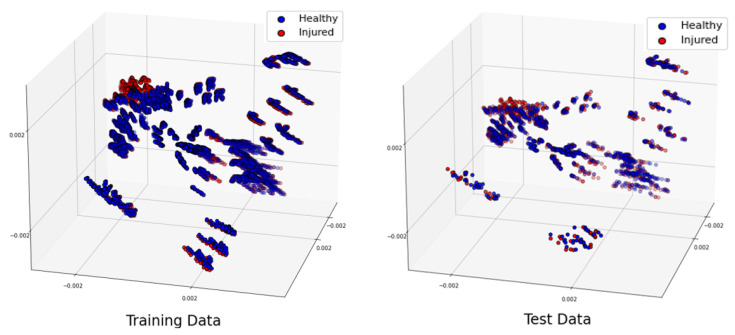
Projection of the 3 most significant eigenvectors of the large dataset: training dataset (**left**) and test dataset (**right**).

To conduct a further study, let us shuffle all the samples we generated for different locations 20 times. Let us remind the reader that our total number of samples was 4464. We separated 3350 samples for training and 1114 samples for testing. The distribution of the training and test datasets for the trial is shown in Figure 10. We repeated this classification for 10 trials, and the accuracy was always higher than 99.91% when using a linear kernel, and the hyperparameter C= 600,000,000. The confusion matrix for the trial is shown in Table 13. The training time for this scenario was 39.2 seconds, whereas the test was performed in real time.

**Figure 11:**
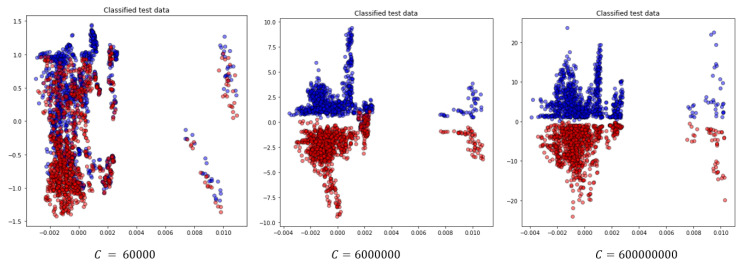
Projection of the two most significant eigenvectors of classified test data for the random test case for different choices for the *C* parameter.

Further, we examine the effect of the *C* hyperparameter on the classification for this scenario. To do this, we vary its value and present the results in Table 14 and Figure 11. We observe that increasing *C* reduces the classification error on the training dataset by allowing a smaller margin, which classifies all training points correctly. Conversely, a smaller *C* encourages a larger margin and a simpler decision function, resulting in lower accuracy. It’s important to note that increasing the *C* value results in a less complex model and an easier optimization problem, speeding up the training process. Additionally, it reduces the number of support vectors, which helps lower the overall computational time [30].

## 4. Conclusions

This study serves as a proof of concept for a microwave digital twin prototype for automatic, noninvasive, portable and real-time detection of healthy and injured tendons in the shoulder. For this, we created a generalizable dataset using state-of-the-art numerical modeling and the proposed imaging system considering various parameters: different noise levels, variations in complex permittivity due to dehydration effects and different locations of the phantom in the imaging system. The computing time for generating each sample was less than two minutes with the use of 160 computing cores. This parametric study led us to several important conclusions:

- With flexible numerical modeling, we can construct a realistic dataset for classifying tendon injuries which addresses the challenge of obtaining real-world datasets.
- By incorporating noise levels into the training dataset, we can ensure accurate injury detection under varying noise conditions.
- By accounting for changes in the measured complex permittivity of tissues due to dehydration, we can maintain high accuracy in injury detection. This is a key issue because it takes into account two important characteristics of human patients:
  – The small difference of dielectric properties from one patient to another for the same human tissue.
  – The variability in the synovial fluid (SF) values. We remind the reader that SF accounts for differentiating healthy from injured shoulders.
- Introducing various possible locations of the shoulder within the imaging system is critical for a realistic dataset. By generating datasets for different shifts and rotations, we can ensure high accuracy in classifying test data.
- When different shoulder locations are included in the training dataset, SVM classification achieves real-time detection of rotator cuff tears (RCTs) with 99.91% accuracy, provided that we train with positions close to those in the test set. We found empirically that the training should not have positions further away than 0.5 cm from the test position. If the distance is larger, then new positions have to be included in the training. This study will have to be pursued with statistical assessments once the system is built in order to see what the real dispersion is in the different patient positions with respect to the imaging system.

The next step is to move from the microwave digital twin prototype to the microwave digital twin by making tests using datasets measured from real-world shoulder models.

## Figures and Tables

**Table 1:** Parametric study for detection of RCTs.

Scenario	Description
Noise level	Introducing different noise levels in synthetic data
Error in value of $\varepsilon_{r}$	Dielectric property variations due to dehydration
Localization	Changes in the location of the shoulder
Randomized dataset	Shuffled training and test dataset

**Table 2:** Complex dielectric properties at 1 GHz.

Different Tissues	Value of $\varepsilon_{r}$
Bone cortical	12.4−2.79i
Tendon	45.6−13.66i
Muscle	54.8−17.43i
Skin	40.9−16.17i
SF	68.42−29.12i

**Table 3:** Confusion matrix interpretation.

		Predicted Class	
		Healthy	Injured
Actual Class	Healthy	TP	FN
	Injured	FP	TN

**Table 4:** The noise levels introduced in each set of data. The values are in dB.

Sample Model	Training Dataset	Test Dataset
Healthy	16.6,13,7,4.4,2.3	19,7.7,4.9,2.7,0.9,−0.65
Injured	23,15.3,5.6,3.9,1.38	31,15.2,6.4,4,1.9,0.24

**Table 5:** Number of generated training and test samples for healthy and injured models. We built the datasets with 36 seeds for each noise level.

Sample Model	Training Dataset	Test Dataset
Healthy	36×5=180	36×6=216
Injured	36×5=180	36×6=216

**Table 6:** Classification results for different values of *C* in noise study.

*C*	Acr	Spec	Sens
6000	90.5%	87.9%	93.0%
600,000	95.3%	100%	90.7%
6,000,000	100%	100%	100%

**Table 7:** The value of $\varepsilon_{r}$ at 1 GHz while including the dehydration effect compared with the original values ($hd_{err}=0$).

Tissue	${\mathrm{hd}}_{\mathrm{err}}=-9\%$	Original Values Based on Table 2	${\mathrm{hd}}_{\mathrm{err}}=9\%$
Bone Cortical	11.28−2.5i	12.4−2.79i	13.5−3.04i
Skin	37.2−14.7i	40.9−16.17i	44.58−17.6i
Tendon	41.5−12.43i	45.6−13.66i	49.7−14.88i
Muscle	49.8−15.86i	54.8−17.43i	59.73−19i
SF	61.8−26.3i	68.0−29.0i	74.1−31.6i

**Table 8:** Number of generated training and test samples for healthy and injured models. We built the datasets with 36 seeds for each noise level.

Sample Model	Total Dataset	Training Subset	Test Subset
Healthy	48×7×3=1008	900	108
Injured, 3 groups of SF values	$48\times 7\times 3\times \left[\begin{array}{c} 1\\ 1\\ 1 \end{array}\right]$ = $\left[\begin{array}{c} 1008\\ 1008\\ 1008 \end{array}\right]$	$\left[\begin{array}{c} 900\\ 900\\ 900 \end{array}\right]$	$\left[\begin{array}{c} 108\\ 108\\ 108 \end{array}\right]$

**Table 9:** Classification accuracy for different values dehydration error for C= 6,000,000.

Value of SF	Accuracy
61.8−26.3i	100%
68−29i	100%
74−31i	100%

**Table 10:** Number of generated training and test samples for healthy and injured models for each position of the phantom.

Sample Model	Training Dataset	Test Dataset
Healthy	3×12×4=144	3×12×4=144
Injured	3×3×4×4=144	3×3×4×4=144

**Table 11:** Number of generated training and test samples for healthy and injured models for 31 positions of the shoulder inside the sensing system due to rotation and shifting.

Sample Model	Total Dataset	Training Subset	Test Subset
Healthy	144×31=4464	4320	144
Injured	144×31=4464	4320	144

**Table 12:** Different scenarios for various positions of phantoms in the imaging system.

	Healthy	Injured	Accuracy	Confusion Matrix
1	M2	M2	100%	$\left[\begin{array}{cc} 144 & 0\\ 0 & 144 \end{array}\right]$
2	M2	M4	100%	$\left[\begin{array}{cc} 144 & 0\\ 0 & 144 \end{array}\right]$
4	M7	M3	91.3%	$\left[\begin{array}{cc} 119 & 25\\ 0 & 144 \end{array}\right]$
5	M9	M6	88.54%	$\left[\begin{array}{cc} 111 & 33\\ 0 & 144 \end{array}\right]$

**Table 13:** Confusion matrix for randomized large dataset with C= 600,000,000.

Models	Healthy	Injured
Healthy	1112	2
Injured	0	1114

**Table 14:** The effect of the *C* value in classification for the shuffled large dataset.

*C*	Acr	Spec	Sens	Time (s)
600	57.09%	45.87%	68.31%	332.5
6000	66.38%	66.25%	66.52%	293.6
60,000	85.63%	80.61%	90.66%	264.8
600,000	95.06%	93.36%	96.77%	138.9
6,000,000	97.3%	96.77%	97.85%	67.8
60,000,000	99.55%	99.91%	99.19%	47.8
600,000,000	99.91%	99.82%	100%	39.2

## Data Availability

The data are contained within the article.
